# PRDM16 Enhances Osteoblastogenic RUNX2 via Canonical WNT10b/β-CATENIN Pathway in Testosterone-Treated Hypogonadal Men

**DOI:** 10.3390/biom15010079

**Published:** 2025-01-08

**Authors:** Siresha Bathina, Mia Prado, Virginia Fuenmayor Lopez, Georgia Colleluori, Lina Aguirre, Rui Chen, Dennis T. Villareal, Reina Armamento-Villareal

**Affiliations:** 1 Division of Endocrinology Diabetes and Metabolism, Baylor College of Medicine, Houston, TX 77030, USA; 2 Department of Medicine, Michael E. DeBakey Veterans Affairs (VA) Medical Center, Houston, TX 77030, USA; 3 Department of Medicine, University of New Mexico School of Medicine, Albuquerque, NM 87107, USA; 4 Department of Medicine, New Mexico VA Health Care System, Albuquerque, NM 87107, USA

**Keywords:** PRDM16, RUNX2, osteoblastogenesis, BMD

## Abstract

We previously reported that *PRDM16* mediated the improvement in body composition in testosterone (T)-treated hypogonadal men by shifting adipogenesis to myogenesis. Previous preclinical studies suggest that *Prdm16* regulates *Runx2*, an important osteoblastic transcription factor, expression and activity. However, the changes in *PRDM16*, and other genes/proteins involved in osteoblastogenesis with T therapy in hypogonadal men are unexplored. We investigated the role of PRDM16 in RUNX2 activation by measuring changes in gene expression in peripheral blood monocytes (PBMCs) and proteins in the serum of hypogonadal men after T therapy for 6 months. Likewise, we evaluated changes in the WNT10b—β-CATENIN signaling pathway by gene expression and protein analyses. We found significant increases in *PRDM16* and *RUNX2* expression in PBMCs together with significant increases in serum proteins at 6 months when compared to baseline. There were also increases in gene and protein expressions of WNT10b, and β-CATENIN at 6 months. Furthermore, we found a significant positive correlation between % changes in PRDM16 and WNT10b. Our results suggest that T therapy activates PRDM16, leading to enhanced signaling in the canonical WNT10b—β-CATENIN-RUNX2 pathway, the pathway involved in osteoblastogenesis. The above findings may account for the improvement in bone density and quality in hypogonadal men treated with T.

## 1. Introduction

Aging is associated with bone loss leading to osteoporosis and an increase in the risk of fragility fractures [1]. Contributory to age-related skeletal deterioration is the gradual decline in testosterone (T) levels with advancing age in men [2]. Previous studies have shown that androgen deficiency leads to bone loss and osteoporosis [3,4]. Prior studies from our group showed an increase in bone mineral density (BMD) with T replacement therapy [5]. Improvement in bone quality with T therapy has also been reported by other authors [6,7,8,9]. Data from in vitro studies supported an anabolic effect of androgens in bone; a study by Wiren showed that androgen treatment resulted in osteoblast differentiation, proliferation, matrix production and mineralization [10]. Another study also showed the activation of osteoblast differentiation in T-treated MC3T3-E1 cells [11].

Recently, we reported that the positive regulatory domain binding motif (PRDM16), which is a transcriptional regulator of brown adipose tissue, mediated the improvement in body composition in hypogonadal men treated with T by shifting adipogenesis to myogenesis [12]. On the other hand, the group of Kaneda et al. (2022) showed abnormal cartilage and bone formation in *Mel1/Prdm16* deficiency mice, suggesting the significant role of *Mel1/Prdm16* expression in bone formation [13]. These animals also showed abnormal expression of Runt related transcription factor (*Runx2*). Furthermore, Shull et al. demonstrated a synergistic association between *Prdm16* with osteoblastogenic *Runx2* in zebra fish [14]. Importantly, Wingless-related integration site 10b (Wnt10b), a ligand in the canonical β-catenin signaling pathway associated with expansion of osteoblast and maintenance of adult mesenchymal stem cells (MSCs) [15], was found to be regulated by *Prdm16* transcriptional activity [16]. However, it remains unclear whether T therapy activation of *PRDM16* also has a downstream effect on the skeletal system in hypogonadal men. The objective of this study is to determine if T activation of PRDM16 will result in activation *WNT-β-CATENIN* signaling resulting in enhanced osteoblastogenesis. We hypothesize that the positive effects of T therapy in bone are mediated by *PRDM16—WNT10b-β-CATENIN-RUNX2* cascade activation leading to enhanced osteogenesis in hypogonadal men.

## 2. Materials and Methods

### 2.1. Study Design and Participants

This study is a secondary analysis of the longitudinal data, and samples are collected from a previously completed single-arm open-label clinical trial (NCT01378299) investigating the effect of *CYP19A1 gene* polymorphisms on the response to T therapy in men with hypogonadism. The details on the study design, inclusion and exclusion criteria, and intervention have been published elsewhere [5]. Briefly, this study enrolled men, between 40 and 75 years of age with an average fasting total T from 2 blood draws taken between 8 and 11 a.m., at least 30 min apart, of <300 ng/dL. These subjects had no medical problems that may prevent them from completing the study. Exclusion criteria included (1) treatment g medications affecting bone metabolism (e.g., bisphosphonates, teriparatide, denosumab, glucocorticoids, sex steroid compounds, selective estrogen receptor modulators, androgen deprivation therapy, and anticonvulsants); (2) intake of finasteride; (3) osteoporosis or history of fragility fractures; (4) diseases affecting bone metabolism, such as hyperparathyroidism, uncontrolled or untreated hyperthyroidism, significant renal impairment (creatinine of >1.5 mg/dL), and chronic liver disease; (5) history of prostate or breast cancer; and (6) untreated obstructive sleep apnea.

The participants were male veterans who were patients at the Endocrine, Urology and Primary Care Clinics of the New Mexico Veterans Administration Health Care System (NMVAHCS) and Michael E. DeBakey Veterans Affairs Medical Center (MEDVAMC). These subjects were recruited using flyers, posters, or letters to physicians, and informed consent was obtained from each subject in writing. The protocol was approved by the Institutional Review Board of the University of New Mexico School of Medicine (protocol number HRPO-11-139) and the Baylor College of Medicine (protocol number H-34812); the study was conducted in accordance with guidelines in the Declaration of Helsinki for the ethical treatment of human subjects.

### 2.2. Testosterone Therapy

Intramuscularly injected testosterone (cypionate) was initiated at a dose of 200 mg every 2 weeks and adjusted to a target serum T level between 500 and 800 ng/dL. T therapy was administered for 18 months. A total of 51 subjects at NMVAHCS underwent self-injections; 38 received injections from the study team only, while 2 started with the study team but later opted for self-injection. At MEDVAMC, 5 subjects received injections from the study team, while 10 chose self-injections. Adjustments in T doses were made based on serum T and hematocrit levels and the incidence of symptoms, and they were accomplished by increments or decrements of 50 mg. A reduction in the dose was carried out for participants who developed a hematocrit of >52%. Repeat T measurements were performed 2 months after a change in dose, including a repeat hematocrit for those with elevated hematocrit. Otherwise, T measurements were performed at baseline, 3, 6, 12 and 18 months. After the 3rd year of the study, with a directive from the FDA, the target T level was changed to 300–600 ng/dL. This change affected the data in the last 6 months for 16 subjects at NMVAHCS and all 15 subjects at MEDVAMC. Nevertheless, comparing T levels at different timepoints showed no significant differences between those affected and those not affected by the change in target T level, except at 6M, where levels were higher for those affected. Measurements of prostate specific antigen (PSA), hematocrit, lipid profile, and liver enzymes at baseline, 3, 6, 12, and 18 months were performed as part of safety monitoring. Body mass index (BMI) was calculated as weight (kg) divided by the square of the height (m^2^). Height and weight were measured using a standard stadiometer and weighing scale, respectively.

### 2.3. Areal BMD

The areal BMD (aBMD) of the lumbar spine, proximal left femur and whole body (WBBMD) were performed at baseline, 6, 12, and 18 months by dual energy X-ray absorptiometry (DXA) using Hologic Discovery (Hologic Inc., Bedford, MA, USA). Femoral regions of interest include the total hip and femoral neck. The coefficients of variation (CV) at our center were ~1.1% for the lumbar spine and ~1.2% for the proximal femur [17].

### 2.4. Gene Expression Studies

#### 2.4.1. Peripheral Blood Mononuclear Cells

Gene expression studies on *PRDM16*, *WNT10b*, *β-CATENIN*, *RUNX2* from PBMCs was performed by real-time quantitative polymerase chain reactions at BL and 6M. We used a RiboPure Blood (Invitrogen, #AM1928) kit for RNA extraction from PBMCs; later, 200ng of RNA was used for retro transcription into cDNA and performed using Superscript VILO Master Mix (Invitrogen, Carlsbad, CA, USA) in triplicates following kit instructions accordingly. FAM-labeled TaqMan gene expression assays (Applied Biosystem, College Station, TX, USA) were carried out for *PRDM16* (Assay ID: Hs00223161_m1), *WNT10b* (Assay ID: Hs00559664_m1), *β-CATENIN* (Assay ID: Hs00355045_m1), and *RUNX2* (Assay ID: Hs1047973_m1); we used VIC-labeled TaqMan housekeeping 18S (Assay ID: Hs03928990_g1) for gene expression assay and TaqMan Universal Master Mix following the manufacturer’s protocol.

#### 2.4.2. Relative Quantification

We quantified ΔΔCT by comparing gene expression of the treated sample with gene expression of human control total RNA (Applied Biosystem #4307281) and finally adjusted for housekeeping gene expression. We used Quant Studio Design & Analysis Software 1.3.1 for data analysis.

### 2.5. Biochemical Analysis

The following were measured using enzyme linked immunosorbent assay (ELISA) kits PRDM16 Human Elisa kit (Biomatik, EKN52948, Wilmington, DE, USA), Human protein Wnt-10b ELISA kit (My BioSource, MBS9428495, San Deigo, CA, USA), Human β-catenin ELISA kit (My BioSource, MBS266009, San Deigo, CA, USA), RUNX2 Immunoassay kit (My BioSource, MBS452519, San Deigo, CA, USA). The CVs for the above assays in our laboratory are <10%. We performed a longitudinal analysis of gene and protein expressions on the limited number of samples available: *n* = 22 for PBMC and *n* = 38 for serum assessments. Please see the (Supplementary Table S1) for details of kits, chemicals, and instrumentation used in this study.

### 2.6. Statistical Analysis

Previous results from our lab suggested maximal effect of T therapy at 6M. Hence, we chose to study only baseline (BL) and the corresponding 6months (6M) samples. The data were analyzed by Two-tailed Student’s paired or unpaired t test. All analyses were performed using Prism 9.0 (GraphPad, San Diego, CA, USA). A *p* value of ≤0.05 is considered statistically significant. All data are presented as mean ± SEM in the figures and mean ± SD in the table.

## 3. Results

### 3.1. Clinical and Serum Biochemical Parameters at Baseline and After 6 Months of T Therapy

There were 105 men who participated in the original study. However, we only had 40 subjects with serum samples available for analysis. Data on BMD changes in these patients showing improvement in spine BMD with T therapy have been reported elsewhere [18]. Table 1 shows the changes in hormonal profile, and osteoblastogenic and bone turnover markers in the forty men with available baseline (BL) and 6-month (6M) samples of serum are included in the analysis. There were significant increases in T (BL: 258.6 ± 90.34 ng/dL vs. 6M: 578.3 ± 241.7 ng/dL, *p* = 0.001), E2 (BL: 15.69 ± 6.06 pg/mL vs. 6M: 39.4 ± 21.2 pg/mL, *p* = 0.001), and estradiol/testosterone ratio (E/T) (BL: 0.70 ± 90.38 vs. 6M: 71.3 ± 40, *p* = 0.001) (Table 1) at 6 months when compared to baseline. There were significant reductions in markers of bone turnover (osteocalcin and CTX) at 6 months of T therapy.

Next, we explored the gene and protein machinery that may be involved in the significant improvement in bone density and quality with T therapy observed in our [18] and prior clinical studies [19].

Table 1 above illustrates a comparative analysis of clinical and serum biochemical markers before and after 6 months of T therapy. BMI: Body mass index, CTX: C terminal telopeptide of type 1 collagen, OCN: Osteocalcin. PRDM16—Positive regulatory domain binding protein, WNT-10b—Wnt family member 10b, RUNX2—Runt related transcription factor2 in serum samples of hypogonadal men with T therapy. Values are shown as mean ± SD. Bolded *p* values are statistically significant when compared to baseline.

### 3.2. T Therapy Enhanced Gene Expression of PRDM16 and WNT10b—β-Catenin-RUNX2 Signaling Pathway in the PBMC

**Figure 1:**
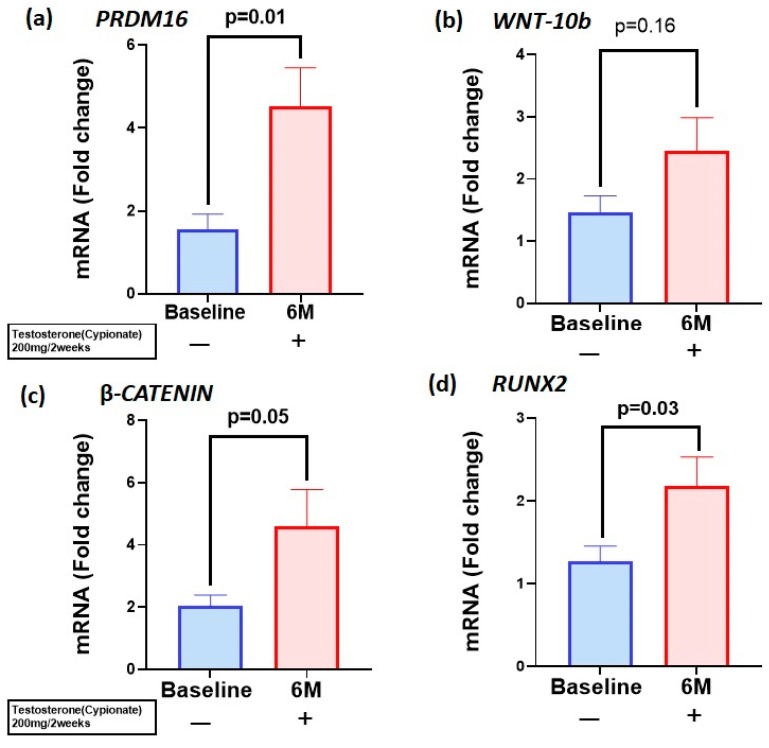
T therapy enhanced mRNA levels of *PRDM16* and osteogenic markers in peripheral blood monocytes (PBMCs): mRNA levels of (**a**) *PRDM16* (**b**) *WNT10b* (**c**) *β-CATENIN* and (**d**) *RUNX2* were increased in PBMCs after 6 months of T therapy in hypogonadal men, which were significant except for *WNT10b*. Data are shown as Mean ± SEM. A *p* value of ≤0.05 is considered significant.

We evaluated the expression of osteoblastogenic gene machinery in the PBMCs of T-treated hypogonadal men. Given the inaccessibility of human bone tissues, we used PBMCs as a surrogate in the examination of osteogenic markers. Results of gene expression studies (Figure 1a) showed that the significant upregulation in *PRDM16* (BL: 1.8 ± 0.42 vs. 6M: 4.5 ± 0.92, *p* < 0.01) was accompanied by significant increases in β-*CATENIN* (BL: 2.02 ± 0.36 vs. 6M: 4.6 ± 1.2, *p* = 0.05), (Figure 1c) and *RUNX2* (BL: 1.32 ± 0.20 vs. 6M: 2.2 ± 0.33, *p* = 0.03) (Figure 1d) after 6 months of T therapy. *WNT10b* levels also increased, although not significantly (BL: 1.46 ± 0.26 vs. 6M: 2.3 ± 0.54, *p* = 0.10) (Figure 1b).

### 3.3. T Therapy Increased Protein Levels of PRDM16, Canonical Markers WNT-10b and β-Catenin and Osteoblastogenic Marker RUNX2

**Figure 2:**
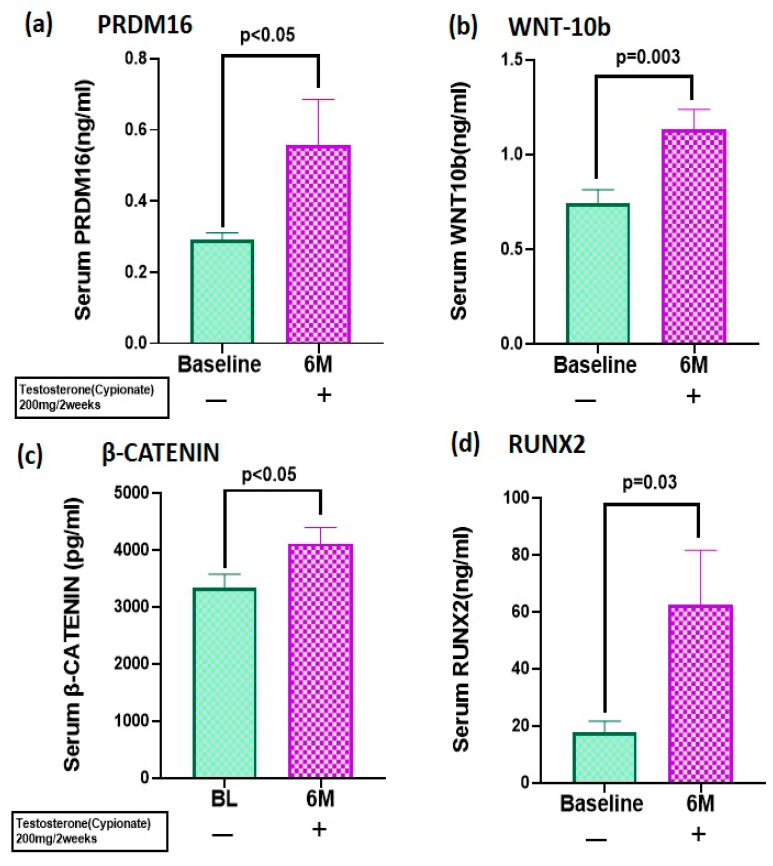
T therapy enhanced osteogenesis via canonical pathway. Protein levels of (**a**) PRDM16 (**b**) WNT10b (**c**) β-CATENIN and (**d**) RUNX2 were significantly increased in serum after 6 months of T therapy in hypogonadal men. Data shown as Mean ± SEM. A *p* value of ≤0.05 is considered significant.

We measured the corresponding proteins in the serum of the genes examined in Section 3.2. There were significant increases in protein levels of PRDM16 (BL: 0.30 ± 0.11 ng/mL vs. 6M: 0.55 ± 0.72 ng/mL, *p* = 0.04) (Figure 2a), WNT10b (BL: 0.74 ± 0.41 ng/mL vs. 6M: 1.14 ± 0.58 ng/mL, *p* < 0.01) (Figure 2b), β-catenin BL: 3164 ± 1100 pg/mL vs. 6M: 3994 ± 1610 pg/mL, *p* < 0.05) (Figure 2c), and RUNX2 protein (BL: 27.8 ± 33.8 ng/mL vs. 6M: 67.7 ± 103.5 ng/mL, *p* = 0.03) (Figure 2d) after 6 months of T therapy.

### 3.4. Correlation Studies

Correlation analysis between molecular markers of bone showed a significant positive correlation between changes in PRDM16 and WNT10b (r = 0.52; *p* = 0.02) in the serum. There were no other correlations found.

## 4. Discussion

Our results showed that T therapy upregulated *PRDM16* gene expression and enhanced *WNT10b*—*β-CATENIN* signaling, resulting in upregulation of *RUNX2*, which is the main transcription factor regulating osteoblastogenesis. These changes in gene expression were accompanied by increased protein production in the serum and likely account for the increase in BMD and improvement in bone quality in T-treated hypogonadal men [18].

Our group previously reported that PRDM16 mediated the improvement in body composition in hypogonadal men treated with T by shifting adipogenesis to myogenesis [12]. Since PRDM16 has also been reported to influence bone and cartilage development [13], we investigated its role in the skeletal response to T therapy, the molecular mechanism behind the changes in bone with T therapy [20] being less clear to date. Previous preclinical studies reported the role of PRDM16 in the conversion of skeletal myoblasts to adipose tissue browning [21,22,23]. Using *Mel1/Prdm16*-deficient mice, the recent studies of Kaneda et al. [13] suggested another important role of *Prdm16*, i.e., in bone development by regulation of osteoblastogenesis and chondrocyte differentiation. These investigators observed that mice deficient in PRDM16 had an abnormal facial skeleton and shortening of long tubular bones [24]. Moreover, studies by Ding et al. showed that Prdm16 knockdown by morpholine disrupted the formation of the craniofacial skeleton in zebra fish [25]. Furthermore, another study by Shull et al. reported [14] that loss of *Prdm16* decreases the expression of both cartilage marker (*sox9a*) and bone markers (*runx2a*) in zebra fish. These preclinical studies also demonstrated that loss of *Prdm16* causes changes in the transcriptomic profile of osteoblastogenic marker *Runx2*. More importantly, data from preclinical studies also demonstrated that *Runx2^(−/−)^*-deficient mice lack osteoblasts and bone formation [26,27]. Consistent with the above findings of a relationship between PRDM16 and RUNX2, our study showed that the significant increase in *Prdm16* was associated with a significantly enhanced expression of osteoblastogenic marker *Runx2* both in PBMCs and serum after 6months of T therapy.

We next studied the pathway involved in the upregulation of RUNX2. Despite the crucial roles of both canonical and noncanonical Wnt signaling in bone remodeling [28], we opted to explore the canonical Wnt pathway for two reasons. Firstly, this pathway promotes bone formation via β-catenin [29]-mediated activation of master osteoblastogenic transcription factor RUNX2, which drives mesenchymal cells to osteogenic lineage [30]. Secondly, preclinical studies identified a significant role of *Prdm16* upstream of Wnt/β-Catenin to balance transcriptional activity during craniofacial chondrogenesis [16]. Since, in our study, PRDM16 was significantly increased in serum and PBMCs, we hypothesized that enhanced *PRDM16* might have a role in osteoblastogenic transcription factor *RUNX2*. For this reason, we next measured the genes *WNT10b* and *β-catenin* in PBMC and in the serum. We found increases in *WNT10b* and *β-CATENIN* gene expression in the PBMCs which was significant for *β-catenin.* We also found a significant increase in protein levels of both in the serum, suggesting that the activation of PRDM16 initiated a signaling cascade through the canonical *WNT10b*—*β-CATENIN* pathway resulting in enhanced *RUNX2* activation with T therapy.

**Figure 3:**
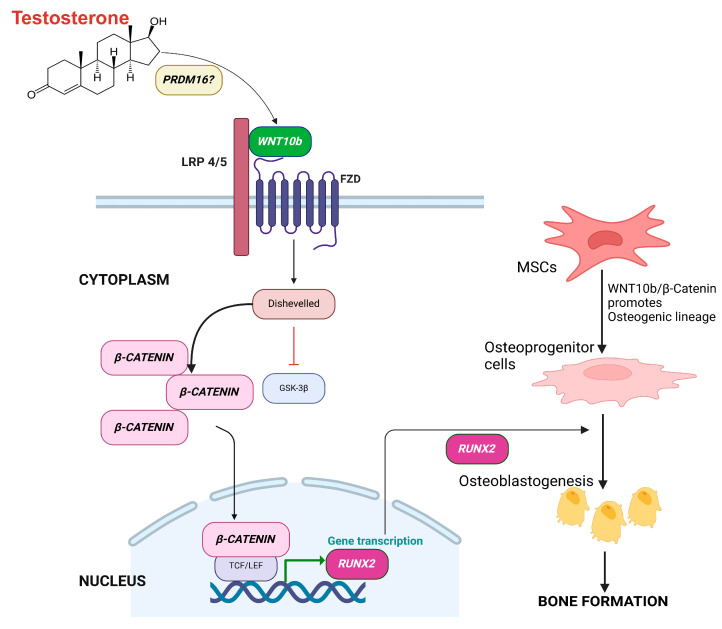
The above figure illustrates the hypothesized molecular mechanism behind the positive effects of *PRDM16* on bone in T-treated hypogonadal men. We hypothesize enhanced *PRDM16* activates canonical *WNT10b* in downstream mechanism by unknown targets which bind to low-density lipoprotein receptor (LRP4/5) and frizzled receptor (FZD) leading to activation of β-catenin in cytoplasm. This molecule enters the nucleus and activates the transcription of osteoblastogenic *RUNX2*. Thus, activation of *PRDM16* initiates a signaling cascade through the canonical pathway resulting in enhanced osteoblastogenesis with T therapy.

Our study provides genomic evidence for a mechanism by which *PRDM16* promotes bone formation through *WNT10b*—*β-CATENIN* pathway leading to activation of *RUNX2*, resulting in commitment of MSCs to the osteogenic lineage, a well-recognized mechanism for osteoblastogenesis and bone formation [31,32,33]. Though how *PRDM16* activates *WNT-10b* is unknown (Figure 3), the activated *WNT10b* binds to seven-pass transmembrane frizzled (FZD) receptor and its co-receptor the low-density lipoprotein receptor-related protein -5 or -6 (LRP5/6) and leads to dimerization of two receptors, causing *β-CATENIN* accumulation. *β-CATENIN* then moves to the nucleus and initiates transcription of *RUNX2* and ultimately bone formation [16].

Despite the increase in the molecular markers suggesting enhanced osteoblastogenesis, there is a decrease in serum osteocalcin, a marker of bone formation. There is also a reduction in CTX, a marker of bone resorption. These findings are consistent with T studies with bone as an outcome [34,35]. The exact role of androgens on the male skeletal metabolism is less clear since T, the key androgen, is also the main source of E2 resulting from aromatase activity in men, and E2 has been established as the primary regulator of bone in men [36,37]. Both androgen and estrogen receptors are found in osteoblasts, osteoclasts, and mesenchymal stromal cells, with the latter transforming into osteoblasts [38,39], adding to the difficulty in teasing out the effect of T distinctively from that of E2 on the male skeleton. Estradiol, for the most part, is an antiresorptive acting to decrease increased bone resorption in patients with hypogonadism. To what extent does the anabolic effect of androgens on bone influence the skeleton separately from E2 is unclear. It is possible that the timing of sample collection may fail to capture the enhanced osteoblastic effect from T. In a study by Wang et al., T administration by patch or gel in different doses for 6 months resulted in an increase in markers of bone formation (osteocalcin, type 1 procollagen and bone alkaline phosphatase) at 3 months followed by a decrease to baseline at 6 months [40]. By contrast, urine N-telopeptide, a marker of bone resorption, was significantly reduced starting at 30 days in the highest dose of T gel and continued to be suppressed until the end of the study. We only measured our bone markers at baseline and 6 months, so we may have missed the anabolic window for bone formation markers. Since both resorption and formation are coupled, ultimately, bone formation is also reduced, and that is what is observed here. On the other hand, there is little or no doubt that androgens contribute to bone size because of their stimulatory effect on periosteal apposition, resulting in bigger and wider bones in men compared to women [17].

The strengths of our study include the following: (1) novelty, as this is the first study to implicate the role of PRDM16 in the molecular mechanism behind the positive effects of T therapy in vivo on bone in hypogonadal men; and (2) our study is the first to explore gene and protein machinery involved in the enhanced osteoblastogenesis in men with hypogonadism given T therapy. However, this study presents several limitations. Firstly, it is a secondary analysis of samples from our prior clinical trial; hence, we have a limited number of samples, *n* = 22 for PBMC and *n* = 38 for protein samples, and these results need to be confirmed in a bigger trial. Secondly, given the inaccessibility of human bone tissues, we used PBMCs as a surrogate in the examination of osteogenic markers. However, the use of PBMCs to investigate events in bone has been carried out in prior studies. In a study on subjects with bone tumors and benign bone tissue, the changes in *β-CATENIN* gene expression in bone tissue parallel that of the changes in the PBMCs [41]. Moreover, a study of young healthy men and women showed that *RUNX2* gene expression in PBMCs and MSCs are highly correlated with BMD in both genders [42].

In summary, our findings provide evidence of a novel downstream regulatory role of *PRDM16* in the activation of *WNT10b/β-CATENIN* transcriptional activity in T-treated hypogonadal men. These events lead to activation of *RUNX2* and commitment of MSCs to the osteogenic lineage leading to improvement in bone density and quality with T therapy. Given the limited sample size in our study, confirmation in a larger trial is needed to validate our findings.

## Figures and Tables

**Table 1:** Clinical and serum biochemical parameters.

	Baseline	After 6 Months	*p* Value
Age	59.8 ± 8.5	60.1 ± 8.7	0.86
BMI	32.1 ± 5.1	32.2 ± 4.9	0.90
Testosterone(T)	258.6 ± 90.34	578.3 ± 241.7	**0.001**
Estradiol	15.69 ± 6.06	37.39 ± 21.2	**0.001**
E/T	0.70 ± 0.38	71.3 ± 40.1	**0.001**
Osteocalcin (ng/mL)	7.1 ± 5.4	4.9 ± 3.4	**<0.05**
CTX (ng/mL)	0.34 ± 0.20	0.22 ± 0.12	**0.005**
PRDM16 (ng/mL)	0.30 ± 0.11	0.55 ± 0.72	**0.04**
WNT-10b (ng/mL)	0.74 ± 0.41	1.14 ± 0.58	**<0.01**
β-CATENIN (pg/mL)	3164 ± 1170	3994 ± 1610	**<0.05**
RUNX2 (ng/mL)	27.8 ± 33.8	67.7 ± 103.5	**<0.05**

## Data Availability

The datasets used and/or analyzed during the current study are available from the corresponding author on reasonable request.

## Supplementary Materials

The following supporting information can be downloaded at: https://www.mdpi.com/article/10.3390/biom15010079/s1, Table S1, for details of kits, chemicals, and instrumentation used in this study.
