# Supplementary material for: PRDM16 Enhances Osteoblastogenic RUNX2 via Canonical WNT10b/β-CATENIN Pathway in Testosterone-Treated Hypogonadal Men
Source: Biomolecules. 2025 Jan 8;15(1):79. doi: 10.3390/biom15010079 (PMC11764227; doi:10.3390/biom15010079)
Supplement: Supplementary file 1 [file biomolecules-15-00079-s001.zip › biomolecules-3396984-supplementary.pdf]

KEY RESOURCES TABLE:

REAGENT/RESOURCE	SOURCE	IDENTIFIER
**Chemicals, Peptides, Metabolites, and Recombinant Proteins**		
RNALater	ThermoFisher	Cat# AM7020
HPLC-grade water	ThermoFisher	Cat# W64
Testosterone-2H3	Sigma-Aldrich	Cat# T2655
2-Mercaptoethanol	Sigma-Aldrich	Cat# M6250
Halt Protease Inhibitor Cocktail (100X)	ThermoFisher	Cat# 78430
HEPES	Biotechne	Car# 7365-45-9
PBS	Corning	Cat# 21-040
**Critical Commercial Assays/Machines**		
RNeasy Plus Universal Mini Kit	QIAGEN	Cat# AM1928
TaqMan Universal PCR Master Mix	ThermoFisher	Cat# 4304437
SuperScript VILO cDNA kit	ThermoFischer	Cat# P5644825
96 well plate for PCR	Applied Biosystems	Cat# N8010560
FastPrep 24–5G homogenizer	MP Biomedicals	Cat # 1606-1026
Nanodrop and Bioanalyzer 2100	Agilent Technologies	Cat# P 275
Real Time PCR system	Applied Biosystem-	Cat # F272520135
Accuwash	ThermoFisher	Cat# 888-7482AF
Digital incubator	Wards science	Cat # 03211213
Spectra max Ab plus	Molecular devices	Cat# ABP 00603
Human PRDM16 Elisa kit	Biomatik	Cat# EKN52948
Human Wnt-10b ELISA kit	My Biosource	Cat# MBS9428495t
Human β-catenin ELISA kit	My Biosource	Cat# MBS266009
Human RUNX2 Immunoassay kit	My Biosource	Cat# MBS452519
**Oligonucleotides**		
Taqman FAM Probe PRDM16	ThermoFisher	Hs00223161_m1
Taqman FAM Probe Wnt10b	ThermoFisher	Hs00559664_m1
Taqman FAM Probe β-catenin	ThermoFisher	Hs00355045_m1
Taqman FAM Probe Runx2	ThermoFisher	Hs1047973_m1
**Software and Algorithms**		
Quantstudio design and analysis software 1.3.1	ThermoFisher	N/A
Endnote version 21	Clarivate Analytics	N/A
Biorender software	Toronto, Ontario, Canada	N/A
Graph pad prism software 9.0	Dotmatics, CA, USA	N/A
SAS version 9.4	SAS institute	N/A
